# Development of in vitro osteoporosis model in minipig proximal humerus and femur: validation in histological and biomechanical study

**DOI:** 10.1186/s13018-023-04102-y

**Published:** 2023-08-22

**Authors:** Jeonghyeon Seo, Hyojune Kim, Joongkee Min, Yongwoo Kim, In-Ho Jeon, Darry D’Lima, Kyoung Hwan Koh

**Affiliations:** 1 Department of Orthopedic Surgery, Areumcheil Hospital, Seoul, Republic of Korea; 2 Department of Orthopedic Surgery, Daejeon Eulji Medical Center, Eulji University School of Medicine, Daejeon, Republic of Korea; 3 Asan Medical Center, Convergence Medicine Research Center, Computerized Tomography Core, Seoul, Republic of Korea; 4 Department of Molecular Medicine, Scripps Research, La Jolla, CA USA; 5 Shiley Center for Orthopaedic Research and Education at Scripps Clinic, Scripps Health, San Diego, CA USA; 6 Department of Orthopedic Surgery, Asan Medical Center, University of Ulsan College of Medicine, 88 Olympic-Ro 43-Gil Songpa-Gu, Seoul, 05505 Republic of Korea

**Keywords:** Osteoporosis, Bone mineral density, Cortical thickness, EDTA, DEXA, Micro-computed tomography, Suture anchor, Biomechanical analysis, Minipig

## Abstract

**Background:**

The minipig has been used for research in various fields of medicine, even in orthopedics. Though previous studies have already suggested other methods to create osteoporotic bone, those methods had some disadvantages for taking time and efforts. Therefore, we aimed to generate osteoporotic proximal humerus and proximal femur of minipig using EDTA solution and validate their properties through dual energy X-ray absorptiometry (DEXA), micro-CT study, histological and biomechanical ways.

**Methods:**

Six minipigs were used. Out of a total of 12 proximal humerus (PH) and 12 proximal femurs (PF), 6 PH and 6 PF were used as the decalcified group and the opposite side as the non-decalcified group. In vitro decalcification with Ca-chelating agents (0.5 M EDTA solution, pH 7.4) was used. Area BMD (aBMD) was measured using DEXA, Volumetric BMD (vBMD), and microstructure were measured using micro-CT. Universal testing machine was used to measure ultimate load to failure (ULTF). Each group was compared using two types of suture anchors (all-suture anchor, ASA, and conventional screw type anchor, CA).

**Results:**

There was a significant difference in aBMD and cortical thickness (aBMD: decalcified, 0.433 ± 0.073 g/cm^2^, undecalcified, 0.962 ± 0.123 g/cm^2^, *p *< 0.001; cortical thickness: decalcified, 0.33 ± 0.34 mm, undecalcified, 1.61 ± 0.45 mm, *p *< 0.001). In the case of ASA, the ULTF was significantly lower in the decalcified group (decalcified: 176.6 ± 74.2 N, non-decalcified: 307.7 ± 116.5 N, *p *= 0.003). In the case of CA, there was no significant difference (decalcified: 265.1 ± 96.0 N, undecalcified: 289.4 ± 114.5 N, *p *= 0.578).

**Conclusion:**

We demonstrated that decalcification with EDTA solution significantly decreased aBMD, vBMD, and cortical thickness. Decalcified minipig bone using EDTA resulted in similar biomechanical properties as osteoporotic human bone with respect to anchor pull-out.

## Background

Osteoporosis is a prevalent and debilitating disease characterized by decreased bone mineral density (BMD) and increased bone fragility, leading to an increased risk of fractures. The development of in vitro animal models for osteoporosis research has proven to be crucial for understanding the underlying mechanisms of the disease and for evaluating new therapeutic strategies. Since many patients undergoing surgery using anchors have osteoporosis, anchor related experiments for osteoporotic bones are necessary and have been attempted [1, 2]. However, it is not easy to obtain osteoporotic cadavers with similar BMD values.

It is most preferable to use human cadaver for biomechanical test, but there are problems such as not being easy to obtain and expensive. As a substitute for human cadaver, several different animals were tried for conducting anchor experiments [3]. Bovine and ovine humeri are not suitable for suture anchor testing. It was because that anchor experiments using bovine humeri showed too strong load to failure compared to human humeri, and in experiments using ovine, some anchors showed significantly lower failure loads compared to human humeri [3]. There are several anchor experiments using pigs [4, 5]. In the case of a mature pig, the weight can reach up to 200 ~ 300 kg [5, 6], and the mechanical strength of pig bones is much stronger than that of humans. The load to failure of anchor is much higher when using porcine humerus than when using human cadaver [4, 5]. There are many studies using minipig in the medical field, cardiology [7], endocrinology [8], pharmacology [9]. In the field of orthopedic surgery, an experiment using a minipig was also conducted in spine pedicle screw fixation [10].

In medical research, there are several ways to artificially create osteoporotic bones. There are methods using ovariectomy or dietary ca restriction to implement osteoporotic bone in animal experiments, but they have the disadvantage of being time consuming, expensive, and labor-intensive [11]. Attempts are being made to compensate for these shortcomings. An attempt was made to create osteoporotic bone using EDTA from the pig spine, and BMD was reduced and significant changes were observed in biomechanical tests [12].

Therefore, the aim of this study was to generate osteoporotic proximal humerus (PH) and proximal femur (PF) bones of minipigs using EDTA solution and validate their properties through dual energy X-ray absorptiometry (DEXA), micro-CT scan, histological, and biomechanical methods. We used an in vitro decalcification method to decrease the bone mineral density through Ca-chelating chemical agent that altered the BMD and biomechanical properties to an extent similar to osteoporotic proximal humerus and femur. First, using DEXA and micro-CT, the changes in area BMD (aBMD), volumetric BMD (vBMD) and cortical thickness (greater tuberosity and greater trochanter of the proximal humerus and proximal femur) by decalcification were measured. Second, 3D microarchitecture was confirmed using micro-CT, and histomorphological property was evaluated. Finally, the mechanical properties of decalcified bone and undecalcified bone were compared through pull out test of two types of anchors (conventional screw and all-suture anchor) for proximal humerus and proximal femur.

## Methods

### Preparation of proximal humerus and proximal femur

**Fig. 1:**
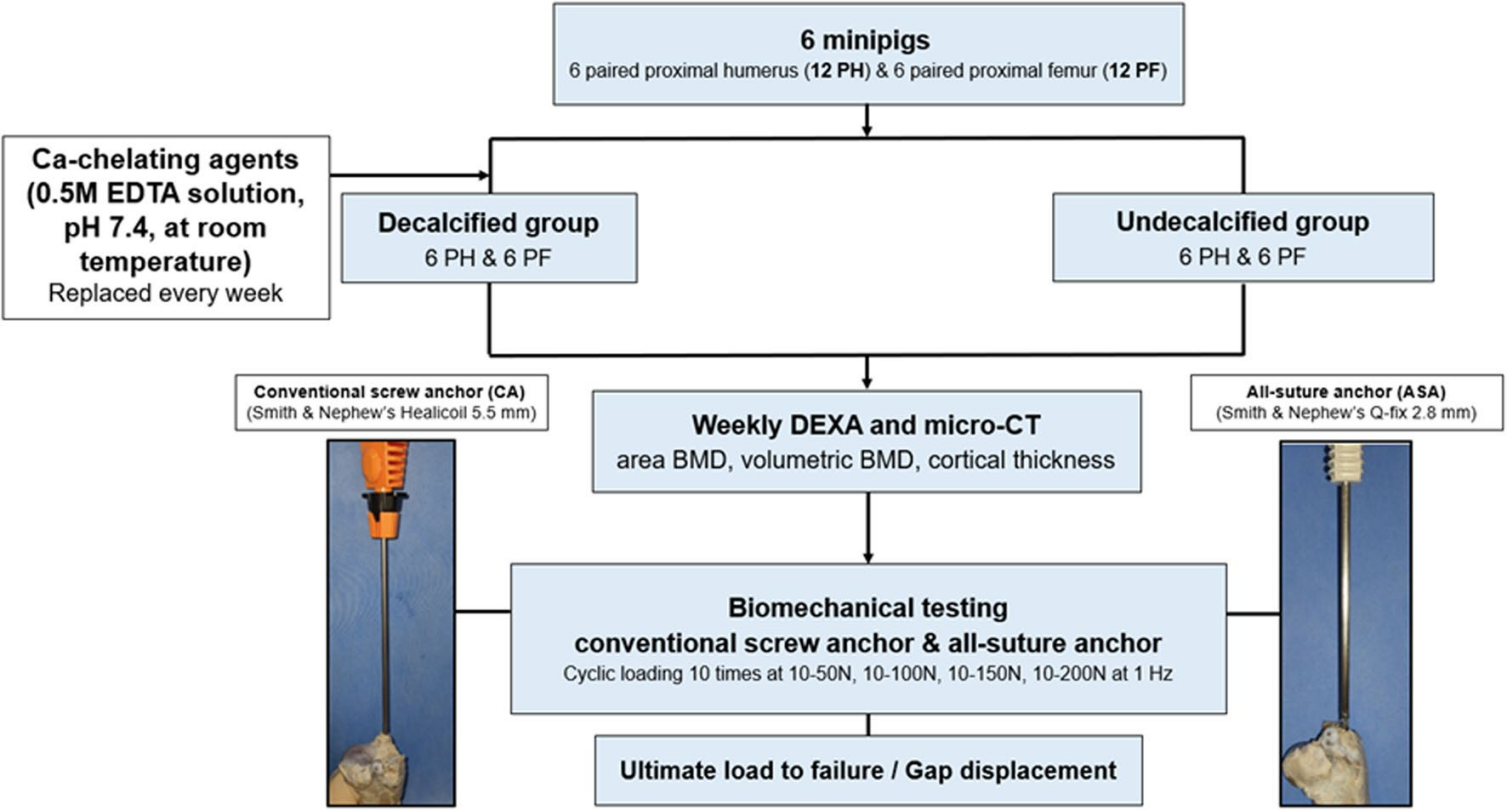
Study design. PH, proximal humerus; PF, proximal femur; DEXA, Dual energy X-ray absorptiometry

This study was performed using 6 paired fresh-frozen proximal humerus and femur harvested from two male and four female minipigs (weight 40–50 kg). All minipigs were killed for other purposes, and there were no medical conditions that could affect bone quality. All specimens were removed from surrounding muscle tissue, ligament tissue, and periosteum, and immersed in 10% formalin (Sigma Cemical Co.) for 24 h after harvest. Among 6 paired humeri and 6 paired femurs, the ones on the right were used as the declacified group and the ones on the left side were used as the non-decalcified group (Fig. 1).

### Decalcification procedure

In the case of the decalcification group, after 10% formalin treatment, each bone was immersed in a separate bottle with 500 ml of 0.5 M EDTA solution (pH 7.4; Sigma) at room temperature. The EDTA solution was replaced with a new one every week. The humerus and femur were subjected to weekly DEXA and micro-CT scans to measure area BMD, volumetric BMD and cortical thickness.

### Dual energy X-ray absorptiometry

Area BMD analyses were performed with a high-resolution DXA analyzer (iNSiGHT VET DXA, Osteosys, Korea). Before weekly measurement, calibration was performed using Ca-HA phantom. The greater tuberosity of the proximal humerus and greater trochanter of proximal femur was designated as a region of interest and photographed. It was assumed that osteoporosis occurred when the area BMD reached about 50% of the initial value, and when it reached the expected level, it was stored as an osteoporotic group in 10% formalin solution.

### Micro-computed tomography

**Fig. 2:**
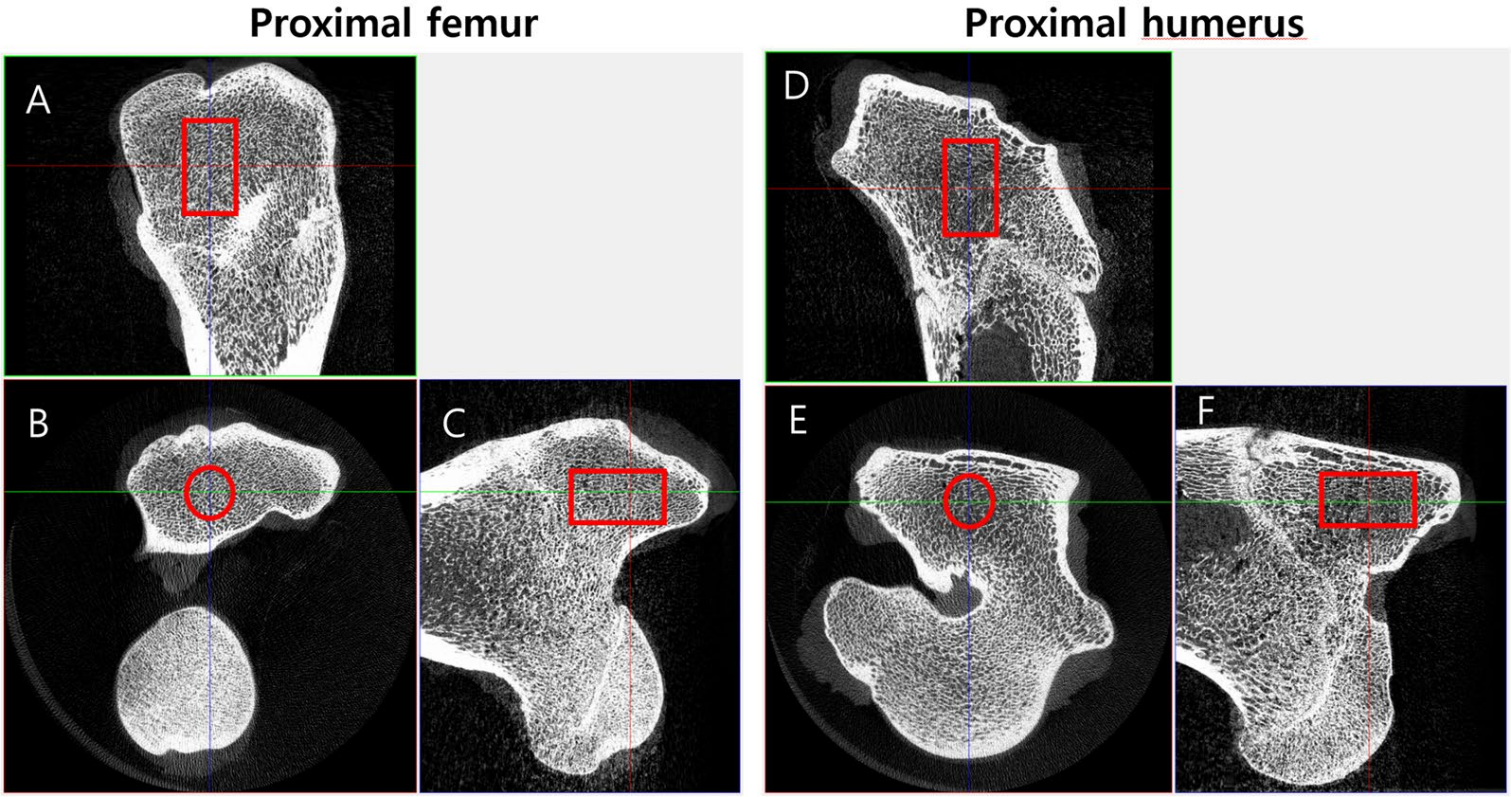
Location of the ROI in micro-CT scan. The ROI was designated 10 mm high, starting at a depth of about 5 mm from the cortex of the greater trochanter of the proximal femur and greater tuberosity of the proximal humerus in sagittal view (**A** and **D**) and coronal view (**C**, **F**), and it was designated as a circle (Ø 5 mm) around the central axis of the head in axial view (**B** and **E**)

All specimens were scanned using a high-definition micro-CT scanner SkyScan 1176 (SkyScan 1176, Bruker-microCT, Kontich, Belgium). The parameters used were 80 kV, 313 µA, 180° rotations, a copper + aluminum filter and a 0.7° rotation step, resulting in an image with a 35.47 µm voxel size. Images of all specimens were reconstructed using NRecon software (NRecon v.1.6.10.4, Bruker-microCT). A 3D evaluation of bone mineral density was performed using CTAn (CTAn v.1.15.4 + (64-bit), Bruker-microCT). Region of interest ROI (Ø 5 mm × 10 mm) was assigned to the greater trochanter of the proximal femur and the greater tuberosity of the proximal humerus (Fig. 2). After then, the resulting ROIs were analyzed. The three-dimensional trabecular microstructure was reconstructed using Mimics research software (Mimics research v. 20.0, Materialise). The trabecular morphometric indices: Percent bone volume (BV/TV), trabecular thickness (Tb. Th.), trabecular number (Tb. N.), and trabecular separation (Tb. Sp.) were analyzed within the defined ROI. Cortical thickness was measured using RadiAnt DICOM Viewer (Medixant. Version 2022.1) for the cortex at the anchor insertion site.

### Biomechanical test using two types of anchors: conventional screw anchor, and all-suture anchor

**Fig. 3:**
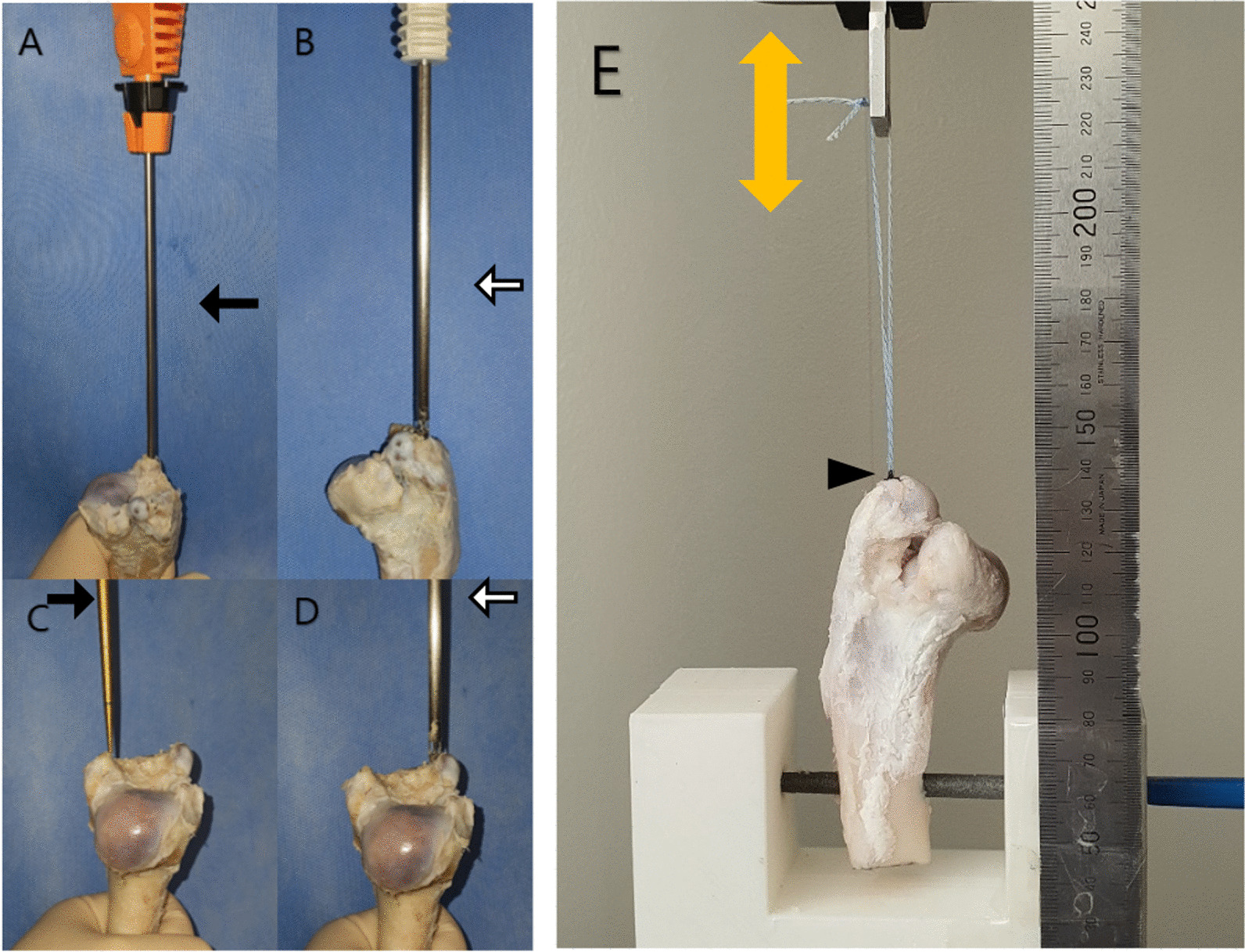
The setting of biomechanical test. Conventional screw anchor (Healicoil PK anchor) (**A** and **C**) was inserted vertically into the posterior part (arrow) of the greater tuberosity of proximal humerus and femur and all-suture anchor (Q-fix anchor) (**B** and **D**) was inserted vertically into its anterior part (empty arrow). By marking the point (arrow head), where the anchor was inserted, the displaced position was confirmed and the moving distance was measured (**E**)

Conventional screw type anchor (CA, Smith & Nephew's Healicoil PK 5.5 mm) and all-suture type anchor (ASA, Q-fix, 2.8 mm) were used. The max diameter of CA is 5.5 mm, and when ASA is deployed in the subcortical layer, it expands to 5.5 mm and is fixed. The anchor was inserted perpendicularly to the GT of the proximal humerus and proximal femur. ASA was inserted into the anterior side of GT, and CA was inserted into the posterior side of GT. After insertion, the mechanical strength of anchor fixation was tested at a 90 degree vertical direction (Fig. 2, Model ST-1001; SALT, Incheon, Republic of Korea). After applying a preload of 10 N, cyclic loading was performed 10 times at 10 ~ 50 N, 10 ~ 100 N, 10 ~ 150 N, and 10 ~ 200 N (1 mm/s). Gap displacement was measured at the end of each cyclic loading. The validated software ImageJ (version 1.53 k; Rasband, W.S., ImageJ, U. S. National Institutes of Health, Bethesda, Maryland, USA) was used for the measurement of the displacement. [13], if the anchors survived the last cycle of testing, pull out force and mode to failure were measured by traction at 1 mm/s (Fig. 3).

### Histologic evaluation

After the biomechanical test, the specimens were cut off (approximately 10 × 10 × 5 mm) at the midpoint between the anterior anchor insertion site and the posterior anchor insertion site. Both decalcified and nondecalcified specimens were used for histologic examination. Specimens were stained with H & E for observation of histological characteristics. The sections were examined by light microscopy.

### Statistical analysis

Statistical analysis was performed to compare the aBMD, cortical thickness, ULTF, and gap displacement between the decalcified and undecalcified groups. The paired t test was used to compare the aBMD, vBMD before decalcification (12 specimens) and after decalcification (12 specimens). The unpaired t test was used to compare undecalcified group (12 specimens) and decalcified group (12 specimens): aBMD, vBMD, cortical thickness, Tb.Th (trabecular thickness), Tb.N (trabecular number), Tb.Sp (trabecular separation), BS(bone surface)/BV(bone volume), BV/TV(total volume), TBPf(trabecular pattern factor), SMI(structure model index). Statistically significance was set at 5% as the threshold. SPSS version 25 was used for all statistical analysis.

## Results

### Evaluation of gross morphology in undecalcified and decalcified model

**Fig. 4:**
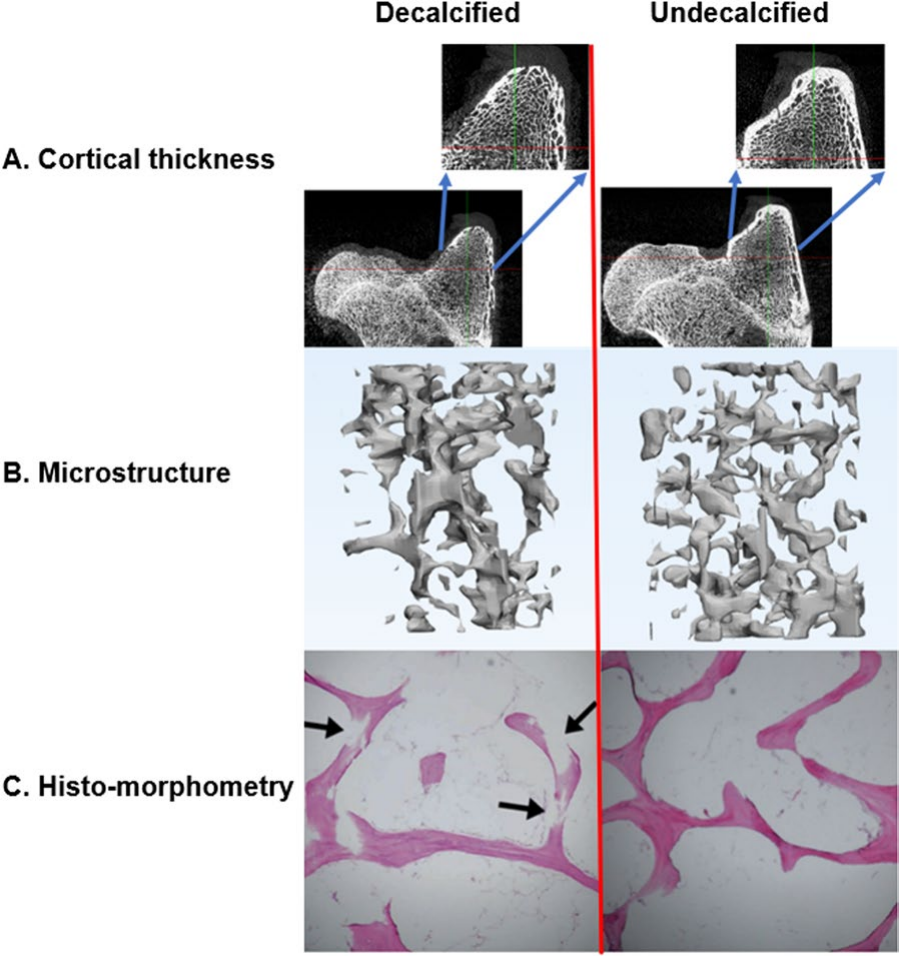
Comparison between decalcified and undecalcified bone model in micro-CT and histomorphometry. The cortical thickness of greater tuberosity of decalcified proximal humerus was obviously thinner than the cortical thickness of undecalcified (**A**). **B** is the 3D reconstructed images of undecalcified and decalcified trabecular bone using Mimics research software. **C** is the histomorphometric changes from undecalcified trabecular bone to decalcified trabecular bone. Discontinuity of trabecular structure occurs after decalcification (arrow) (H&E stain, 100 × magnification)

Using micro-CT and histomorphologic examination, changes in cortical thickness and intra-medullary bony structure were demonstrated after 3 weeks of decalcification with EDTA. Cortical thickness of greater tuberosity of PH and PF in evaluation of gross morphology were substantially thinned (Fig. 3). The intramedullary bony structure was loosely formed in micro-CT 3D reconstructed images, and discontinuity of intra-medullary bony structure was evident in histologic sections (Fig. 4).

### Serial changes in aBMD and cortical thickness after applying EDTA until 3 weeks

**Fig. 5:**
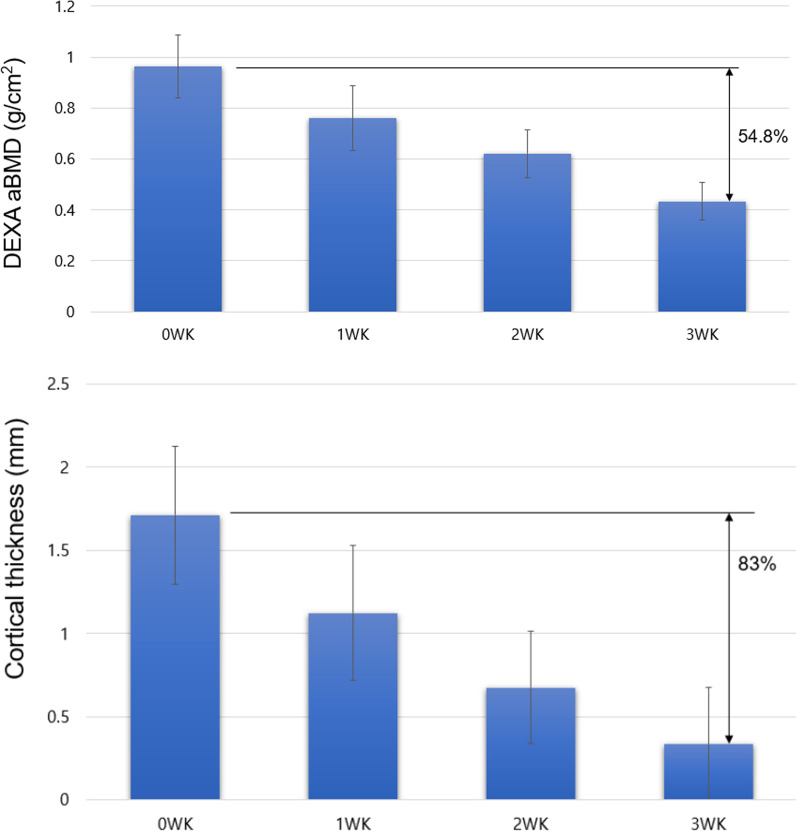
The serial reduction in area BMD and cortical thickness by decalcification up to 3 weeks. DEXA, dual energy X-ray absorptiometry; aBMD, area bone mineral density; wk, week

There was no significant difference between the left and right aBMD values ​​before the experiment (0.04 ± 0.04 g/cm^2^). In the decalcified group, the average aBMD before decalcification was 0.96 ± 0.12 g/cm^2^. After 3 weeks of decalcification, the average aBMD was 0.43 ± 0.07 g/cm^2^, with a significant decrease by 54.8% after decalcification (*P* value < 0.001). The average cortex thickness before decalcification was 1.71 ± 0.41 mm, and after decalcification, it was 0.33 ± 0.3 mm (decreased by 83%, Fig. 5).

### Micro-computed tomography scanning (volumetric BMD, micro structure)

vBMD before decalcification (mean ± SD, 481.1 ± 75.1 mg/ cm^3^) was significantly greater than vBMD after decalcification (360.0 ± 115.1 mg/ cm^3^, *P *= 0.003) (Table 1).

**Table 1:** aBMD and vBMD values of proximal humerus and proximal femur after 3 weeks of decalcification

Specimen	DEXA aBMD (g/cm^2^)			μCT vBMD (g/cm^3^)		
	Proximal humerus and femur			Proximal humerus and femur		
	Undecalcificied	Decalcified	Difference (%)	Undecalcificied	Decalcified	Difference (%)
1Rt.H	0.763	0.343	− 55.0	0.390	0.291	− 25.3
1Rt.F	0.735	0.37	− 49.7	0.468	0.432	− 7.8
2Rt.H	0.888	0.415	− 53.3	0.357	0.271	− 24.1
2Rt.F	0.891	0.322	− 63.9	0.457	0.372	− 18.6
3Rt.H	1.032	0.466	− 54.8	0.437	0.364	− 16.7
3Rt.F	0.993	0.522	− 47.4	0.537	0.523	− 2.5
4Rt.H	1.066	0.497	− 53.4	0.464	0.419	− 9.7
4Rt.F	0.928	0.542	− 41.6	0.546	0.264	− 51.7
5Rt.H	1.144	0.468	− 59.1	0.461	0.388	− 16.0
5Rt.F	1.035	0.433	− 58.2	0.653	0.586	− 10.2
6Rt.H	1.118	0.491	− 56.1	0.541	0.175	− 67.7
6Rt.F	0.948	0.332	− 65.0	0.461	0.235	− 49.0
Mean ± SD	0.962 ± 0.123	0.433 ± 0.073	− 54.8 ± 6.3	0.481 ± 0.075	0.360 ± 0.115	− 24.9 ± 18.7
*p* value	< 0.001			0.003		

*H* Proximal humerus, F Proximal femur

### ***Trabecular microstructure (***Table 2***)***

**Table 2:** Microstructural parameters in decalcified and undecalcificed model

	Trabecular microstructure				Significance
	Decalcified bone		Undecalcificed bone		
	Mean	SD	Mean	SD	
BV/TV (%)	30.6	12.2	45.87	7.84	0.002*
BS/BV (1/mm)	19.22	4.81	19.71	2.86	0.771
Tb.Pf (1/mm)	− 3.4	3.69	− 7.21	4.06	0.031*
SMI	0.72	0.72	0.44	0.49	0.287
Tb.Th (mm)	0.19	0.27	0.19	0.32	0.946
Tb.N (1/mm)	1.83	0.54	2.45	0.36	0.005*
Tb.Sp (mm)	0.52	0.3	0.23	0.07	0.007*

*Significant difference compared to the decalcified bone group

When comparing decalcified bone and undecalcified bone, the values of BS/BV, and Tb.Th did not show significant differences. The BV/TV value of the decalcified group was 30.6 ± 12.2%, which was significantly lower than that of the undecalcified group of 45.87 ± 7.84% (*P *= 0.002). The Tb.Pf value of the decalcified group was − 3.4 ± 3.69 (1/mm), which was significantly higher than that of the undecalcified group − 7.21 ± 4.06 (*P *= 0.031). The Tb.N value of the decalcified group was 1.83 ± 0.54 (1/mm), which was significantly lower than that of the undecalcified group of 2.45 ± 0.36 (*P *= 0.005). The Tb.Sp value of the decalcified group was 0.52 ± 0.3 (mm), which was significantly lower than that of the undecalcified group of 0.23 ± 0.07 (*P *= 0.007).

### Biomechanical test (pull out test for two type of anchors)

The ultimate load to failure (ULTF) value obtained by using ASA in the decalcified group was 176 ± 74.2 N, which was significantly lower than 307.7 ± 116.5 N in the undecalcified group (*P *= 0.003). The ULTF value obtained by using CA in the decalcified group was 265.1 ± 96.0 N, compared to 289.4 ± 114.5 N in the non-decalcified group, with no statistically significant difference. Gap displacement did not show a significant difference in both groups for both ASA and CA (Table 3).

**Table 3:** Biomechanical test for Ultimate load to failure (UTLF) and gap displacement in decalcified and undecalcificed bone using two types of anchors

	Bone density				Significance
	Decalcified bone		Undecalcificed bone		
	Mean	SD	Mean	SD	
*Ultimate Load to failure (N)*					
ASA (2.8 mm)	176.6	74.2	307.7	116.5	0.003*
CA (5.5 mm)	265.1	96.0	289.4	114.5	0.578
*Displacement(mm)*					
ASA (2.8 mm)	1.9	1.5	2.7	1.0	0.185
CA (5.5 mm)	1.8	0.9	1.9	1.0	0.681

*ASA* All-suture anchor, *CA* Conventional screw anchor

*Significant difference compared to the decalcified bone group

## Discussion

We generated osteoporotic proximal humerus (PH) and proximal femur (PF) bones of minipigs and measured their properties through dual energy X-ray absorptiometry (DEXA), micro-CT scan, histological, and biomechanical methods. The present study showed that decalcification using EDTA solution significantly decreased aBMD, vBMD, and cortical thickness of the proximal humerus and proximal femur of the minipig. Furthermore, in biomechanical tests, all-suture anchors (ASA) had a significantly lower ultimate load to failure in the decalcified group compared to the non-decalcified group, while there was no significant difference between the two groups in the case of conventional screw type anchors (CA).

Our measured aBMD of the greater tuberosity of the proximal humerus and greater trochanter of the proximal femur in the decalcified model (0.43 ± 0.07 g/cm^2^) and non-decalcified model (0.96 ± 0.12 g/cm^2^) were similar to previously reported human aBMD. This similarity allowed us to find a similar result in the ultimate load to failure of the human anchor biomechanical test in our minipig study. In other studies, aBMD ranged from 0.08 to 0.57 g/cm^2^ (mean 0.44 g/cm^2^) for the proximal humerus GT of individuals aged 59–87 years old [14]. In healthy men aged 40–49 years old, aBMD was reported as 0.543 ± 0.100 g/cm^2^, and for affected side proximal humeri in women aged 70–79 years old, aBMD was reported as 0.351 ± 0.110 g/cm^2^ [15]. However, the vBMD of our osteoporotic model was 360.0 ± 115.1 mg/cm^3^, which was higher than the reported data for human proximal humerus GT (209.3–285.2 mg/cm^3^) [16]. Since vBMD is a measurement of trabecular vBMD using micro-CT, a three-dimensional measuring device, vBMD reflects only trabecular change (24.9% decrease). aBMD is a result measured using a 2-dimensional x-ray device using DEXA and physically reflects both cortical and trabecular changes. The aBMD change (− 54.8%) can be said to be the result of reflecting both cortical (thickness) change (83% decrease) and trabecular (vBMD) change (24.9% decrease).

Secondly, we were able to develop an osteoporotic model in a shorter time compared to previous studies. In the experimental group, compared to non-decalcified group, aBMD was decreased by 54.8%, and vBMD was by 24.9%. In a study by Lee et al., osteoporotic spine was developed using EDTA solution for 8 weeks, resulting in a decrease of aBMD by 48.9% and vBMD by 80% [12]. Compared to this study, our methods resulted in a greater decrease in aBMD in a shorter term (3 weeks), but with relatively less decrease in vBMD. This difference could be due to the maturity of the pig used in our study compared to that used in Lee et al.'s study. Additionally, Lee et al. used pig spines, whereas we used minipig proximal humerus and proximal femur which have significant differences in cortical and trabecular structure.

In terms of trabecular microstructure, BV/TV (%) was 33.3% lower (*p *= 0.002) in the decalcified group than in the control group, while Tb.Pf (1/mm) showed a 52.8% higher value in the decalcified group (*p *< 0.05). Tb.Pf. is a value for the relation of convex to concave structural elements. Tb.Pf. is a very sensitive parameter for the detection of changes in trabecular bone structure. In osteoporotic bone, loss of connectedness results in predominance of convex structures, which is reflected in the Tb. Pf. Lower Tb. Pf values indicate greater connectivity [17]. In our study, the non-decalcified group showed lower Pb.Pf values. As could be seen in the histologic specimen, concave penetration was increased. It can be assumed that structural stability has decreased. Tb.N was 25% lower in the experimental group, and Tb.Sp was 126% higher in the experimental group. SMI is an index representing the characteristics of cancellous bone structure and is expressed as a number from 0 to 3. An object consisting purely of plates would have a structure model index of 0 and an object consisting purely of rods would have a structure model index of 3 [18]. It showed a 63% higher aspect than the control group, so it can be seen that it is close to a rod-shaped structure, but it was not significant.

In a study that measured the microarchitecture of the human proximal humerus, Tb.Th. osteoporotic bone (0.114 mm) was lower than that of healthy bone (0.138 mm). Osteoporotic bone has a Tb.Sp. of 0.705 mm, while healthy bone has a slightly lower Tb.Sp. of 0.694 mm [19]. In our experiment, Tb.Th. was 0.19 mm in decalcified bone and 0.19 mm in undecalcified bone, and Tb.Sp. was 0.52 mm and 0.23 mm, respectively, which was different from that reported for human humerus. According to the study on trabecular bone microarchitecture in male osteoporosis, BV/TV, Tb. Th. had no significant correlation with the occurrence of vertebral fracture. However, decreasing Tb. N. and increasing Tb. Sp. increased the odds ratio of fracture [20]. In our study, there were significant changes in Tb.N. and Tb.Sp. after decalcification, and no significant change in Tb. Th. A previous study also used EDTA to decalcify porcine vertebrae [12] and reported significant changes in Tb.N and Tb.Sp, but a small reduction (− 13.52%) in Th.Th [12]. These relatively minor differences compared to our results could be explained by differences in bone specimens (vertebrae vs humerus and femur) and decalcification period (2 months vs. 3 weeks).

Minipig cortical thickness before decalcification was higher (1.71 ± 0.41 mm) than that reported for human humerus (0.1–1.2 mm) [1]. These differences may have been due to differences between species and the fact that the human proximal humerus is a non-weight bearing bone while the minipig's proximal humerus is weight bearing. After decalcification, cortical thickness decreased by 83%, a relatively greater decrease than that in the trabecular structure. In our experiment, soaking in EDTA likely induced more rapid removal of calcium from the outer cortex resulting in greater cortical osteoporosis. After decalcification, minipig mean cortical thickness (0.33 ± 0.34 mm) was within the range reported for humans (0.1–1.2 mm) by a previous biomechanical study, investigating the mechanical properties of all-suture anchors in human cadaveric greater tuberosity of proximal humerus (aged 50–73 years old) [1].

Lastly, the biomechanical values of CA and ASA in the minipig were consistent with previously reported data. In cadaveric study, UTLF was ranged from 130 to 350 N, which is similar to our findings [1]. This suggests that the use of this minipig model may be considered for other biomechanical studies. Moreover, ULTF of the ASA was significantly lower in the decalcified group compared with the non-decalcified group. Suture anchors would be strong enough if it can withstand a pullout force of 250 N [21]. In the case of CA, both the decalcified group (265.1 N) and the non-decalcified group (289.4 N) showed an average of 250 N or more. In the case of ASA, the non-decalcified group (307.7 N) showed more than 250 N, but the decalcified group (176.6 N) did not reach 250 N. This suggests that the risk of pullout is higher when using ASA in osteoporotic bone.

## Limitations

First, our decalcification method using EDTA was effective for decreasing aBMD, but trabecular vBMD did not decrease as much as aBMD due to the greater effect of EDTA on the outer cortical layer. Second, this experiment was conducted in vitro. Although the decalcification process using EDTA solution was different from the in vivo physiologic progression of osteoporosis, our results of mechanical anchor pullout in porcine cadavers were consistent with those reported for humans. Additionally, the finding that cortical thickness, aBMD, and vBMD could be modulated to a given target makes this process useful to simulate osteoporotic models for further study.

## Conclusion

Our study demonstrated that decalcification with EDTA solution significantly decreased aBMD, vBMD, and cortical thickness of the proximal humerus and proximal femur of the minipig. Decalcified minipig bone using EDTA resulted in similar biomechanical properties as osteoporotic human bone with respect to anchor pull-out, while non-decalcified bone was similar to healthy human bone. These results indicate that osteoporosis could be simulated with EDTA in minipig, and this model could be applied to other biomechanical tests of osteoporotic bone in orthopedics. The mechanical performance of ASA reduces significantly in decalcified bone, suggesting that CA screw type anchors might be indicated over ASA anchors in osteoporotic bone.
